# Novel MNZ-type microwave sensor for testing magnetodielectric materials

**DOI:** 10.1038/s41598-020-73696-8

**Published:** 2020-10-12

**Authors:** Abhishek Kumar Jha, Nicolò Delmonte, Adam Lamecki, Michal Mrozowski, Maurizio Bozzi

**Affiliations:** 1 Department of Microwave and Antenna Engineering, ETI Faculty, Gdańsk University of Technology, Gdańsk, Poland; 2 Department of Electrical, Computer and Biomedical Engineering, University of Pavia, Pavia, Italy

**Keywords:** Energy science and technology, Engineering, Materials science

## Abstract

A novel microwave sensor with the mu-near-zero (MNZ) property is proposed for testing magnetodielectric material at 4.5 GHz. The sensor has a double-layer design consisting of a microstrip line and a metal strip with vias on layers 1 and 2, respectively. The proposed sensor can detect a unit change in relative permittivity and relative permeability with a difference in the operating frequency of 45 MHz and 78 MHz, respectively. The MNZ sensor is fabricated and assembled on two layers of Taconic RF-35 substrate, with thicknesses of 0.51 mm and 1.52 mm, respectively, for the measurement of the sample under test using a vector network analyzer. The dielectric and magnetic properties of two standard dielectric materials (Taconic CER-10 and Rogers TMM13i) and of yttrium–gadolinium iron garnet are measured at microwave frequencies. The results are found to be in good agreement with the values available in the literature, which shows the applicability of the prototype for sensing of magnetodielectric materials.

## Introduction

Among the various techniques of miniaturizing radio frequency (RF) and microwave circuits, the most fundamental technique is to design the circuits on a dielectric substrate with relatively high permittivity. The size of the microwave device is proportional to the inverse of the square root of the relative permittivity of the substrate material. However, this technique has limitations, mostly arising from incompatible impedance matching with other circuits and the adverse effect on radiation efficiency and bandwidth. Device miniaturization using ferrite materials is a better option, though only for frequencies up to 300 MHz, as the loss becomes very high above the VHF range^1^. Magnetodielectric materials with dielectric constant and relative permeability greater than unity are an attractive alternative to ferrites and dielectrics for miniaturization, as they give one more degree of freedom that helps in the design of high-frequency circuits^2^. The promising results that have emerged from studies of the advantages and limitations of designing RF and microwave devices using magnetodielectric materials, instead of ferrites or pure dielectric materials, open up new opportunities for the development of compact and reconfigurable future generation high-frequency circuits and systems^1–3^. Reducing the size of microwave devices by a factor of the product of the inverse squares of the relative permittivity and permeability, while keeping the wave impedance nearly unaltered (by keeping the ratio of the relative permeability to permittivity constant) is very attractive, with the result that magnetodielectric materials are now being used in electromagnetic interference (EMI) suppression, electromagnetic absorbers, and device reconfigurability^3,4^.

Magnetodielectric materials are usually synthesized with the addition of ferrites to dielectric materials. This is not a straightforward method, and there are many fabrication-related factors that affect their electromagnetic properties on the macroscopic level. For these reasons, precise characterization of such materials is essential^4^. There are two kinds of techniques for characterizing magnetodielectric materials at microwave frequencies: the transmission–reflection type and the resonant type. The parameter extraction procedures using transmission–reflection measurements proposed in the literature are modified or improved versions of an earlier technique^5,6^. However, non-resonant methods that have been suggested in the past, with one exception^7^, fail when the sample size is a multiple of a half-wavelength. Despite this drawback, transmission–reflection techniques are capable of providing coarse values of material parameters over a broadband of RF and microwave frequency. The resonant technology of magnetodielectric characterization is much more accurate, but the results are valid only in a narrow frequency band. Two groups have recently proposed resonant microwave characterization techniques for magnetodielectric materials, based on two electrically small resonators, split ring resonator (SRR)^8^ and the complementary SRR (CSRR)^9^. Open resonators like SRR and CSRR do however have limitations in terms of the quality factor. Closed resonators are preferred for measuring the quality factor. Another vital aspect of the microwave characterization of materials using the resonant technique is the intensity of the electric and magnetic field at the sensing area of the resonator. A higher electromagnetic field provides better sensitivity in the microwave characterization of the material. An example of a highly sensitive miniature resonator is a substrate integrated waveguide (SIW) sensor with an epsilon-near-zero (ENZ) channel^10^. The ENZ channel squeezes and confines the electric field into a comparatively smaller volume, which increases its intensity in the sensing area for dielectric characterization. Recently, an isotropic mu-near-zero (MNZ) media is theoretically proposed, which is capable of concentrating the magnetic field invariant of position and number of the MNZ scatterer^11^.

In this paper, we propose to make use of a mu-near-zero (MNZ) region for the construction of a novel sensor to extract the material parameters of magnetodielectric samples. In a mu-near-zero (MNZ) region, the magnetic field intensity is nearly constant^12^; to our best knowledge, this property has not been explored to date for material characterization. We propose a novel MNZ type of sensor for measuring dielectric and magnetic properties of magnetodielectric materials at the design frequency. The sensor has a double-layer design consisting of a microstrip line where a metal patch is used to create a virtual ground with the help of blind vias. The magnetic field and the electric field are separated in the device by means of blind vias and metal patches. Detailed numerical analysis reveals that this gives a high degree of control in measuring the dielectric and magnetic properties of a magnetodielectric sample. The performance of the sensor is tested by loading various standard dielectric and magnetic samples. Error analysis is performed for positioning and air gap errors during the sample measurement. To demonstrate the practical usefulness of the device, a yttrium gadolinium iron garnet (GdY_2_Fe_5_O_12_) sample, which shows dielectric as well as magnetic properties, is characterized and tested using the proposed sensor. The results show good agreement with the literature.

## Theory of ENZ and MNZ

To introduce the concepts of ENZ and MNZ and to highlight some phenomena associated with them, let us consider a parallel plate waveguide (PPW) viz*.* PPW1 that supports the TEM wave as a dominant mode of wave propagation. The characteristic impedance and propagation constant of PPW1 are given as *Z*_TEM,1_ = *η*_1_*b*/*w*, and *β*_1_ = *ω*(ε_1_*μ*_1_)^0^^.5^, respectively, where *η*_1_ = (*μ*_1_/ε_1_)^0.5^ is the intrinsic impedance of the medium between the plates, *b* is the distance between the two plates, *w* is the width of the plates, *ω* is the angular frequency, *ε*_1_ is the permittivity and *μ*_1_ is the permeability of PPW1^13^. Let another parallel plate waveguide viz. PPW2 with the same width but a different height, *h,* and length *l* is connected with two identical PPW1 at both the ends, where the PPW1 serves as the input and output transmission lines. The characteristic impedance and propagation constant of PPW2 are Z_TEM,2_ = *η*_2_*h*/*w* and *β*_2_ = *ω*(*ε*_2_*μ*_2_)^0.5^, where *η*_2_ = (*μ*_2_/*ε*_2_)^0.5^, *ε*_2_ is the permittivity and *μ*_2_ is the permeability of PPW2. Assuming a significant difference in height between PPW1 and PPW2, so that we have a sharp discontinuity^14^, propagation of a wave through the discontinuity (a variable height of two-plate waveguide) is possible when there is an impedance match. If we neglect the shunt admittance at the interface between the mismatched waveguides^15^, the reflection coefficient (S11) and transmission coefficient (S21) can be expressed using the transmission line theory (while assuming time variation, *e*^*jωt*^) and given as^14^:

1 $$\begin{aligned} {\text{S}}11 & = \frac{{ - j\left( {Z_{{\text{TEM,1}}}^{2} - Z_{{\text{TEM,2}}}^{2} } \right)\sin (\beta_{2} l)}}{{2Z_{{\text{TEM,1}}} Z_{{\text{TEM,2}}} \cos (\beta_{2} l) + j\left( {Z_{{\text{TEM,1}}}^{2} + Z_{{\text{TEM,2}}}^{2} } \right)\sin (\beta_{2} l)}} \\ {\text{S}}21 & = \frac{{2Z_{{\text{TEM,1}}} Z_{{\text{TEM,2}}} }}{{2Z_{{\text{TEM,1}}} Z_{{\text{TEM,2}}} \cos (\beta_{2} l) + j\left( {Z_{{\text{TEM,1}}}^{2} + Z_{{\text{TEM,2}}}^{2} } \right)\sin (\beta_{2} l)}} \\ \end{aligned}$$

In order to determine the condition for the transmission, we need to consider only the medium properties and the distance between the two plates. It can be shown from (1) that for ideal case, i.e., zero reflection and full transmission, the characteristic impedance must be matched, i.e., Z_TEM,1_ = Z_TEM,2_, which gives *η*_2_ = *η*_1_*b*/*h*. If the PPW1 is made of standard non-magnetic material having permeability, *μ*_1 =_ *μ*_0_ and permittivity, *ε*_1=_ *ε*_0_*ε*_r_, while PPW2 has permeability *μ*_2_ = *μ*_0_*μ*_eff_ and *ε*_2_ = *ε*_0_*ε*_eff_*,* the effective wave impedance of PPW2 can be given as the ratio of effective permeability *μ*_eff_ and effective permittivity *ε*_eff_ of the medium, i.e., (*μ*_eff_/*ε*_eff_)^0^^.5^ = *b*/*h*(*ε*_r_)^0.5^, where *ε*_r_ is the dielectric constant of the non-magnetic material used for PPW1, *ε*_0_ and *μ*_0_ are representing the permittivity and permeability of free space. From this straightforward expression, we can estimate the two limiting cases: (1) for *h*/*b* → 0, *ε*_eff_*/μ*_eff_ → 0; and (2) for *b*/*h* → 0, *μ*_eff_/*ε*_eff_ → 0. Within the practical limits, it can be understood that when *h* → 0, the effective permittivity of the channel has an ENZ effect—that is, *ε*_eff_ → 0. In contrast, for *b* → 0, the effective permeability of the channel has an MNZ effect: *μ*_eff_ → 0. The consequence of this is that the ENZ channel has a very high impedance, while the MNZ channel has a very low impedance. A simple arrangement of parallel plate discontinuities of varying heights can modulate the effective permittivity and permeability of the medium between the plates. Interestingly, under this condition, the curl of either the electric or the magnetic field vanishes. Despite the presence of the finite electromagnetic field, the first two Maxwell expressions suggest a quasistatic model of wave propagation through the channel. Since the dominant mode of the parallel plate waveguide is TEM, the propagation of the orthogonal electric and magnetic field without phase variation along the direction of propagation through the channel appears as the tunneling effect. The uniform, quasistatic electric field, and magnetic field inside the channel can be utilized for sensing the dielectric and magnetic properties of the test materials.

## Engineering ENZ or MNZ channels in rectangular waveguides

At microwave frequencies, making use of the concept of parallel plate waveguide-based MNZ transition, as described in “Theory of ENZ and MNZ” section, requires additional arrangements. In such methods, artificially engineered metamaterial structures are utilized to fill the channel, which causes the characteristic impedance of the channel region to match that of the waveguide regions. An increase in the characteristic impedance of the channel for ENZ^15^ and the decrease in the impedance of the channel for MNZ^14^ provides ideal transmission characteristics at higher operating frequencies. For example, the artificial material is placed in the channel to satisfy the Drude–Lorentz dispersion model^14^, where the permeability of the material vanishes at the operation frequency. The inclusion of such artificial materials increases the electric field intensity for ENZ^15^ and the magnetic field intensity for MNZ^14^ channels.

However, the tailoring of frequency-based ENZ and MNZ effects using the parallel plate waveguide is not straightforward at microwave frequencies. A better way is to utilize a metallic waveguide^16^ of rectangular cross-section (width *w* and height *b*), which is intrinsically dispersive. For a metallic waveguide filled with a dielectric of relative permittivity *ε*_r_, the effective permittivity for the dominant mode at frequency *f* can be given as *ε*_eff_ = *ε*_r_ − (*c*/2*wf*)^2^, where *c* is the speed of light^17^. For a given dimension of the metallic cavity filled with a known dielectric material, the values of effective permittivity with respect to frequency shows dispersive nature. Below the cutoff frequency of the dominant mode *f*_c_, the value of *ε*_eff_ is a negative number, which reaches zero near *f*_c_ before attaining a positive number above *f*_c_. In other words, it is possible to effectively obtain zero or negative relative permittivity values of the medium inside a rectangular waveguide made of metal and filled with standard dielectric materials, which possesses positive values of relative permittivity, by just varying the width *w* of the rectangular waveguide. It can be easily shown that an air-filled waveguide inherently possesses an ENZ effect near the cut-off frequency of the TE_10_ mode. The greater strength of the electric field in the metallic waveguide is quite useful and serves the purpose of dielectric sensing at microwave frequencies without filling the ENZ channel of the parallel plate waveguide with an artificial inclusion, as in SRRs^10,18^.

Although a rectangular waveguide does not support TEM waves, we can still use the transmission line model, provided we define the characteristic line impedance and propagation constant through an effective medium. As explained in the transmission line analysis, the ENZ effect occurs in a rectangular waveguide near cutoff^17^. The ENZ channel can be formed to allow tunneling across an abrupt transition from *b* to *h* (*b* >> *h*) by selecting the width of the channel so that it operates near cutoff. An MNZ channel is needed to sense magnetic properties. Let us investigate whether we can achieve MNZ conditions (tunneling and a constant phase of the magnetic field along the channel) in a rectangular waveguide by reversing the transition geometry (*b* << *h*).

**Figure 1:**
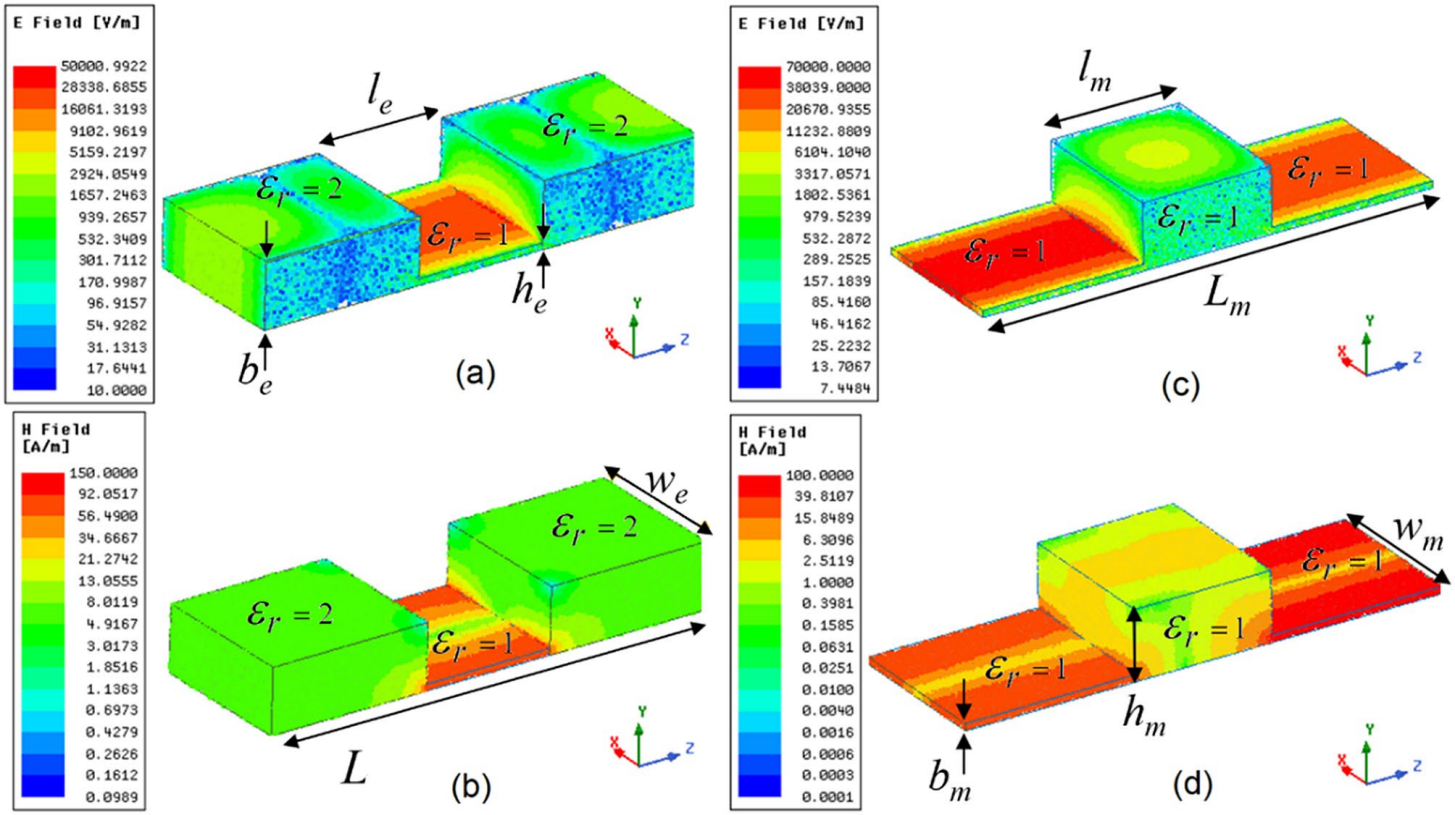
(**a**) Electric field and (**b**) magnetic field distribution in an ENZ metallic waveguide; (**c**) electric field and (**d**) magnetic field in an MNZ waveguide (*b*_e_ = *h*_m_ = 10.16 mm*, h*_e_ = *b*_m_ = 1 mm, *w*_*e*_ = *w*_*m*_ = 22.86 mm, *l*_e_ = *l*_m_ = 20 mm, *L*_*e*_ = *L*_*m*_ = 70 mm) [ANSYS Electromagnetics Suite, Release 2019 R2, https://www.ansys.com/products/electronics].

The dominant mode in a rectangular waveguide is TE_10_. This mode is possessed by one component of the electric field, *E*_y_ and by two components of the magnetic fields, *H*_x_ and *H*_z_ (when propagating in the z-direction), which be written as follows^13^:

2 $$\begin{aligned} H_{z} & = A\cos \left( {\frac{\pi }{{w_{i} }}x} \right)e^{ - j\beta z} , \, E_{y} = - \frac{j\omega \mu \pi }{{k_{c}^{2} w_{i} }}A\sin \left( {\frac{\pi }{{w_{i} }}x} \right)e^{ - j\beta z} \\ H_{x} & = \frac{j\beta \pi }{{k_{c}^{2} w_{i} }}A\sin \left( {\frac{\pi }{{w_{i} }}x} \right)e^{ - j\beta z} \\ \end{aligned}$$

where *β* is the propagation constant, *k*_c_ is the wavenumber at cut-off frequency, and the suffix *i* can be replaced with *e* for ENZ and *m* for MNZ waveguide, as shown in Fig. 1. For a fair comparison, the width (*w*_e_ = *w*_m_), channel length (*l*_e_ = *l*_m_), overall length (*L*_e_ = *L*_m_), and ratio of the height (*b*_e_/*h*_e_ = *h*_m_/*b*_m_) are kept in equal proportion. WR90 waveguides with ENZ and MNZ transitions are simulated using the full-wave numerical solver Ansys HFSS. It should be noted here that the ENZ and MNZ characteristics are observed near the cutoff of the transition geometry. To show this effect, the ENZ waveguide is modeled in such a way that the cutoff frequency of ENZ transition is of higher frequency than the cutoff frequency of the other regions. The single-mode transmission with this imposed condition can be achieved by taking the relative permittivity *ε*_r_ of the transition region (= 1) to be less than the relative permittivity of other regions (= 2), as can be seen in Fig. 1a and b. However, this effect can also be experienced when the cutoff of each region is at the same frequency—i.e., the relative permittivity of each region is the same (= 1), as is shown in Fig. 1c and d. The 3D electric field and magnetic field for ENZ and MNZ waveguides are plotted on the 2D scale and are shown in Fig. 1 at 6.21 GHz and 6.56 GHz, respectively.

**Figure 2:**
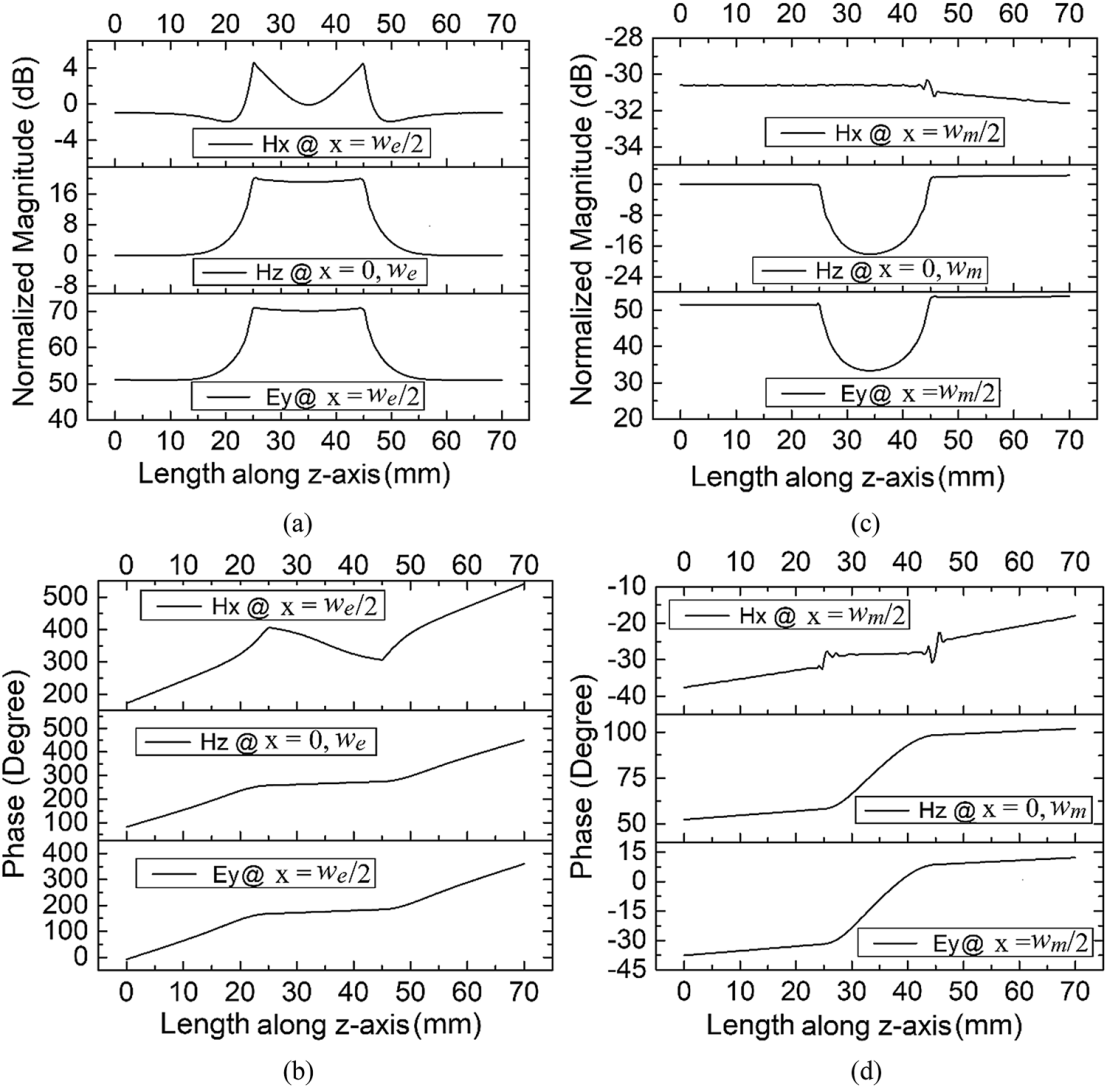
The (**a**) magnitude and (**b**) phase of *E*_y_, *H*_x,_ and *H*_z_ of the ENZ waveguide; the (**c**) magnitude and (**d**) phase of the MNZ waveguide.[OriginLab, 2020b, https://www.originlab.com].

The electric field and magnetic field configurations of a rectangular waveguide with an abrupt junction of height *h*_*e*_ << *b*_*e*_ and length *l*_*e*_ are shown in Fig. 1a and b, respectively, while the abrupt junction of height *h*_*m* >>_ *b*_*m*_ and length *l*_*m*_ are shown in Fig. 1c and d, respectively. It may be noted here that, for *h*_*m*_ >> *b*_*m*_, the upper limit of *h*_*m*_ could be justified by operation of the dominant TE_10_ mode—i.e., *h*_*m*_ < 2*w*_*m*_. Since the rectangular guide possesses only one electric component, it can be observed from Fig. 1a and c that the electric field intensity is amplified in the channel of height *h*_*e*_ << *b*_*e*_, and is reduced in the channel of height *h*_*m*_ >> *b*_*m*_. This difference is not only observed in the magnitude: the phase shows distinctive properties, too. The phase variation along the wave propagation is almost constant in the channel of height *h*_*e*_ << *b*_*e*_, while there appears to be a sinusoidal variation of phase in the channel of height *h*_*m*_ >> *b*_*m*_. Due to the inherited property of possessing the quasistatic, intensified, and uniform electric field throughout the channel—which is only possible with an ENZ medium—the channel of height *h*_*e*_ << *b*_*e*_ can be referred to as an ENZ channel. By analogy, the channel with height *h*_*m*_ >> *b*_*m*_ can be referred to as an MNZ channel. Since the rectangular waveguide in the dominant mode has two components of the magnetic field, it is hard to explain the individual components directly from Fig. 1b and d. For clarity, each component of the electric and magnetic fields for the ENZ and MNZ guides are plotted separately in Fig. 2.

From (1), it can be calculated that (*H*_x_ = 0)_x = 0,*w*_, (*H*_z_ = 0)_x = *w/2*_, and (*E*_y_ = 0)_x = 0,*w*_, hence the electric field and magnetic fields are evaluated at the middle and the endpoints of the width *a* and numerically computed along the length of the waveguide. The magnitude and phase of the *E*_y_, *H*_x_, and *H*_z_ of ENZ and MNZ guides are plotted in Fig. 2a–d, respectively. Since the founding expression for evaluating electromagnetic fields inside a rectangular waveguide operating in the TE_10_ mode is *H*_z_^13^, as can also be observed in (2), the magnitude of the electric and magnetic fields are normalized with the magnitude of *H*_z_ inside the normal waveguide. From Fig. 1a, it appears that the magnitudes of *E*_y_ and *H*_z_ are amplified by nearly 20 dB over the respective input amplitudes, and are constant, while the amplitude of H_x_ is quite weak and not constant inside the ENZ channel. An interesting observation can be made from Fig. 1b, where the phase of *E*_y_ and *H*_z_ begins varying linearly through the waveguide length, while it appears to be constant within the ENZ channel length, and again varies linearly outside the ENZ channel. However, the phase of *H*_x_ is not constant in the ENZ channel. Since the magnitude of *H*_x_ is minimal (less than − 24 dB, as compared with the input amplitude of *H*_z_), the effect of the nonuniform *H*_x_ can be ruled out. In the recent past, intense uniform electric fields with almost zero magnetic fields at x = *w*/2 have been effectively used for the dielectric sensing of materials placed in the ENZ channel^10,18^.

From Fig. 2c and d, it can be observed that the magnitudes of *E*_y_ and *H*_z_ do not experience amplification and that the phases of *E*_y_ and *H*_z_ do not remain constant inside the MNZ channel. If we neglect the higher-order modes at the junctions, the magnitude and phase of *H*_x_ quite interestingly remain constant inside the MNZ channel. Though the amplitude of *H*_x_ is still quite weak compared to the magnitude of *H*_z_ inside the channel, in the ENZ channel, the constant magnitude and uniform phase of the *H*_x_ are not enough to produce the decoupling effect. The amplification of the *H*_x_ is possible after introducing the metamaterials inside the MNZ channel, as has been experimentally verified for parallel plate waveguide in the recent past^14^. However, introducing artificial materials that follow the Drude–Lorentz dispersion model inside the channel would also enhance the magnitude of *H*_z_, increasing the difficulty of the overall design. *Prima facie*, it appears that the sensing of magnetic materials using the MNZ channel is not as simple as dielectric sensing using the ENZ channel of a rectangular waveguide. We can summarize the major limitations of MNZ channels for magnetic sensing: (1) the intensity of the uniform magnetic field *H*_x_ is very weak; (2) unlike with ENZ channels, where the only electric component for dielectric sensing was *E*_y_, the presence of another varying magnetic field *H*_z_ could introduce complications to the magnetic measurement. To conclude, although adjusting the height allows us to create conditions corresponding to an MNZ channel in a rectangular waveguide, the uniform magnetic field component in the channel *H*_x_ is not usable. A new structure is therefore proposed in the next section, which circumvents these limitations. In this new structure, the electric and magnetic fields are decoupled, and the phase of the magnetic field is uniform throughout the sensing region.

## Design of proposed sensor and numerical analysis

The proposed structure combines a microstrip line and a sensing region with blind vias. Periodic blind vias were investigated in the substrate integrated waveguide^19^ (SIW) context, where they were found to separate the electric and magnetic fields and to produce a slow-wave effect. This effect appears in the broadband frequency, where the magnitude and phase change as a function of distance. In a narrow frequency band, blind vias and metal patches are used to decouple the electric and magnetic fields through the resonant effect. The numerical analysis shows the MNZ characteristics of the sensor at the resonating frequency.

### Design of proposed double-layer sensor

**Figure 3:**
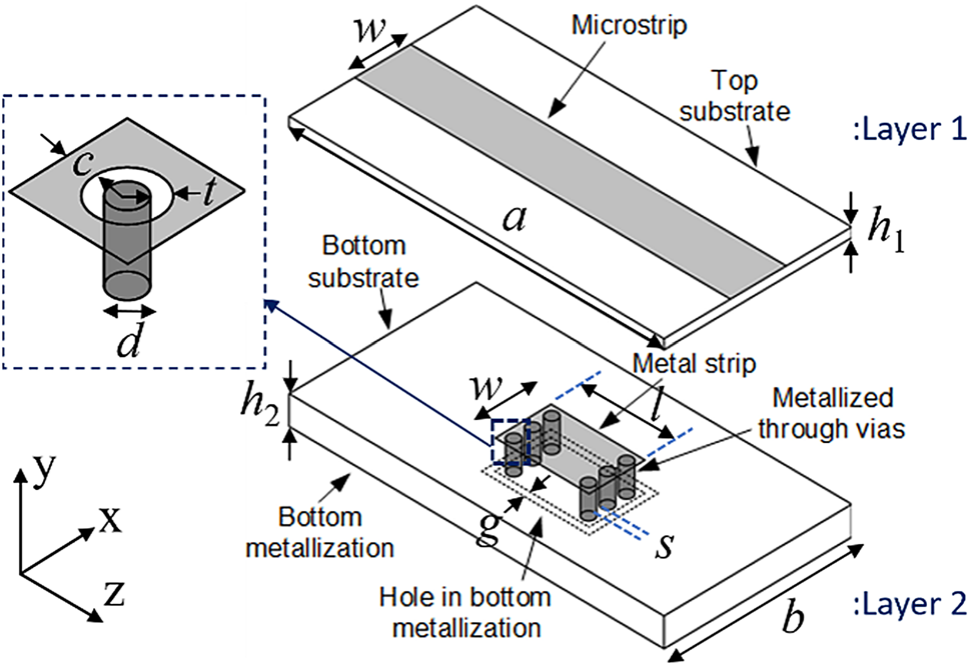
Perspective sketch diagram of a proposed double-layer microwave sensor.[Microsoft Office Professional Plus 2019, https://www.office.com].

The design of the proposed novel MNZ sensor is presented in Fig. 3. The sensor is designed on two Taconic RF-35 substrates (with dielectric constant 3.5 and loss tangent 0.0018 at 1.9 GHz), with heights *h*_1_ for layer1 and *h*_2_ for layer2. Layer1 of the sensor is a simple microstrip line without the ground plane, but the width of the microstrip line, *w*, is chosen as the effective width of a 50 Ω microstrip line of substrate height *h*_1_ + *h*_2_. Layer2 is shown in Fig. 3, and has a metal patch of dimensions *w* by *l* on the top plane; the bottom plane is metalized and serves as the common ground for both layers. A metal patch on the bottom plane of the same dimension as on the top plane is created by etching the ground metallization with thickness *g*. Two rows of three metal vias are created between the top and bottom metal patches. The distance between two vias in the same row is *s*, while the two rows are separated by a distance *l* − 2(*d* + *c* + *t*), i.e., 9.1 mm These metal vias of height *h*_2_ are not directly connected with the metal patch on the top plane: there is a concentric air gap of thickness *t*, as shown in the inset of Fig. 3, and two rows of vias are directly connected through the ground patch. The other ends of the blind vias, which are kept in close proximity with another metal patch buried between layer1 and layer2, produce a confined capacitive region, which makes a larger part of the structure an inductive region at the resonance. Similar arrangements of vias between the top and bottom planes of the substrate integrated waveguide (SIW) has been investigated for decoupling the electric field and magnetic field, which provides the slow-wave effect at the wideband of microwave frequency^19^. The slow-wave effect has been introduced due to the periodicity of the via, where the decoupling of the electric field and its occurrence between the tip of vias and the top plane of SIW is due to the enhanced capacitive effect. However, the magnetic field is present in the whole volume^19^, including the space between the vias; thus technically, both the electric and the magnetic fields are present in the space between the tip of the via and the top plane of the SIW. In contrast to the previous design, the proposed design brings novelty in making both the electric as well as the magnetic fields concentrated at the two physically distant locations. The metallic patch at the top plane of layer2 is electromagnetically coupled to the microstrip line at the top plane of layer1. This metallic patch maintains a concentric separation from the vias that are electrically connected to the metal patch at the bottom plane of layer2. Since the two rows of vias are separated such that they only couple through the metallic patch, the net effect is resonating. At the resonance, the electric field is highly concentrated in the gap *t*, whereas the magnetic field is concentrated in the volume enclosed by the two rows of vias and the two metal patches of layer2. The highly concentrated electric and magnetic fields at these two strategic locations are accessed for sensing through the rectangular slits etched at the bottom plane of layer2. Various design parameters of the proposed double-layer sensor are as follows: *a* = 61 mm, *b* = 25 mm, *c* = 2.3 mm, *d* = 0.6 mm, *g* = 0.4 mm, *h*_1_ = 0.51 mm, *h*_2_ = 1.52 mm, *l* = 15.5 mm, *s* = 0.9 mm, *t* = 0.3 mm, and* w* = 4.8 mm.

### Numerical analysis of the proposed sensor

#### Electric and magnetic fields of sensor

**Figure 4:**
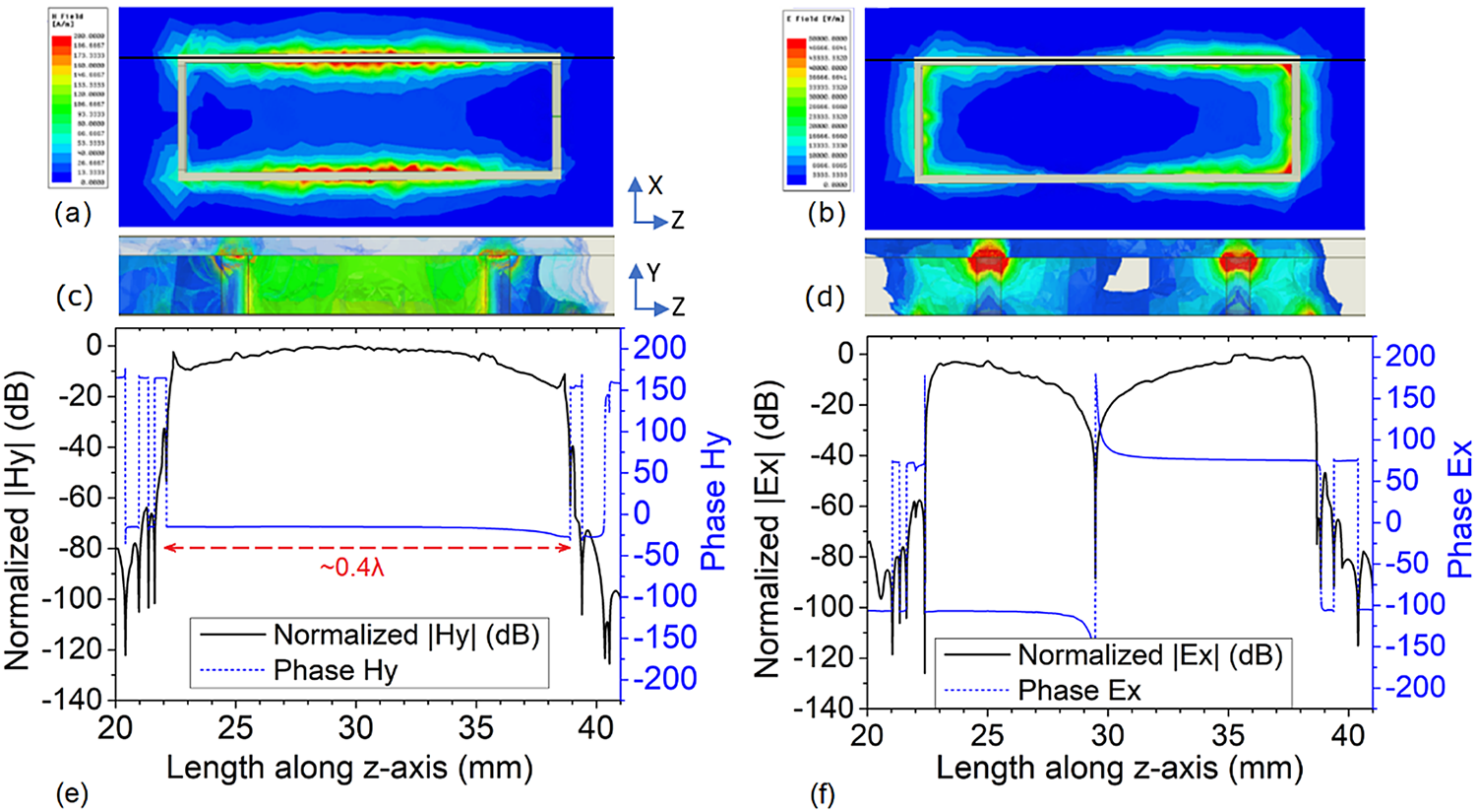
(**a**) Magnetic and (**b**) electric fields in the ground plane, (**c**) magnetic, and (**d**) electric fields x = *b*/2 at 4.5 GHz. The magnitude and phase of (**e**) the *y*-component of the magnetic field and (**f**) the *x*-component of the electric field along the direction of propagation calculated between the slot in ground plane [shown as the black line in (**a**,**b**)] [ANSYS Electromagnetics Suite, Release 2019 R2, https://www.ansys.com/products/electronics; and OriginLab, 2020b, https://www.originlab.com].

The proposed double-layer sensor is numerically tested in the commercial electromagnetic solver Ansys HFSS. The magnetic field and electric field at the resonant frequency (4.5 GHz) are closely observed in the numerical solver, and their intensities are plotted in Fig. 4. From this, it can be observed that the magnetic and electric fields at 4.5 GHz are located in two separate regions. Figure 4a and c confirm that the magnetic field is mainly confined to layer2, surrounded by two rows of blind vias and the two metal patches, whereas Fig. 4b and d confirm the presence of the electric field in close proximity to the vias and the two side edges of the rectangular patch in the ground. The electric field is negligible in the central section. It is thus expected that, if a magnetodielectric sample is placed in the central part, the perturbation in the magnetic field component at *z* = *a*/2 will be only due to the magnetic properties, and there would be almost no change in the magnetic field due to the dielectric properties of the sample. Similar behavior is expected for electric field perturbation when the magnetodielectric sample is placed near the side edges of the rectangular patch in the ground plane. It may be noted here that the small asymmetrical response (not a perfect mirror image at *z* = *a*/2) in the plot of the electric and the magnetic fields, as observed in Fig. 4, is mainly due to the fact that the excitation is applied to one port at a time.

Quantitative analysis (not shown here) demonstrates that the phase of the magnetic field in both layers is uniform between the two rows of vias, while the magnetic field intensity is 3 dB higher in layer2 than in layer1. The magnetic field profile in layer2 confirms the MNZ characteristics of the sensor. However, we have no access to these interior regions for sensing purposes. We can, however, use the fields that leak through the slot in the ground plane to perform measurements. To characterize these leaking fields, the electric and magnetic fields are computed at the mid-point of the slot along the direction of propagation (along the black line in Fig. 4a and b). The electric and magnetic field profiles are shown in Fig. 4e and f. Assuming the quasi-TEM mode in the microstrip, the *y*-component of the electric field and the *x*-component of the magnetic field have a dominant effect inside layer1 and layer2. On the ground plane, at the opening of the slots along the *z*-direction, we find that the *y*-component of the magnetic field and the *x*-component of the electric filed are dominant; they are plotted in Fig. 4e and f, respectively. We observed that the other components of the electric and magnetic field are evanescent and only appear in the vicinity of the sharp discontinuities, e.g., near the vias and slots. We can observe from Fig. 4e that the phase of the magnetic field is uniform along the length of the slot, while the intensity is higher than in layer1. The key idea of the MNZ sensor is to obtain a combination of the intensified and the uniform magnetic field, and the constant phase throughout the sensing region. On the other hand, it is evident from Fig. 4f that the electric field intensity drastically decreases and attains a minimum value at the center of the sensing region; a 180° phase change is also observed. From these observations, it can be concluded that the magnetic field profile of the proposed design preserves the MNZ effect in the sensing region on the ground plane and, therefore, can be effectively used to sense the magnetic material. From Fig. 4b and d, we can see that the intensity of the electric field is maximum near the edge of the rectangular slot on the ground plane; this region is, therefore, suitable for sensing dielectric properties. The sensing of both the electric and the magnetic properties of the material makes this sensor an ideal candidate for the magnetodielectric material sensing at microwave frequencies. As can be seen from Fig. 4d, the electric field is quite intense in the concentric gap *t*. The value of the resonant frequency dominates this parameter, which can then be easily tuned to give the sensor different resonant frequencies.

#### Independent sensing of dielectric and magnetic properties

**Figure 5:**
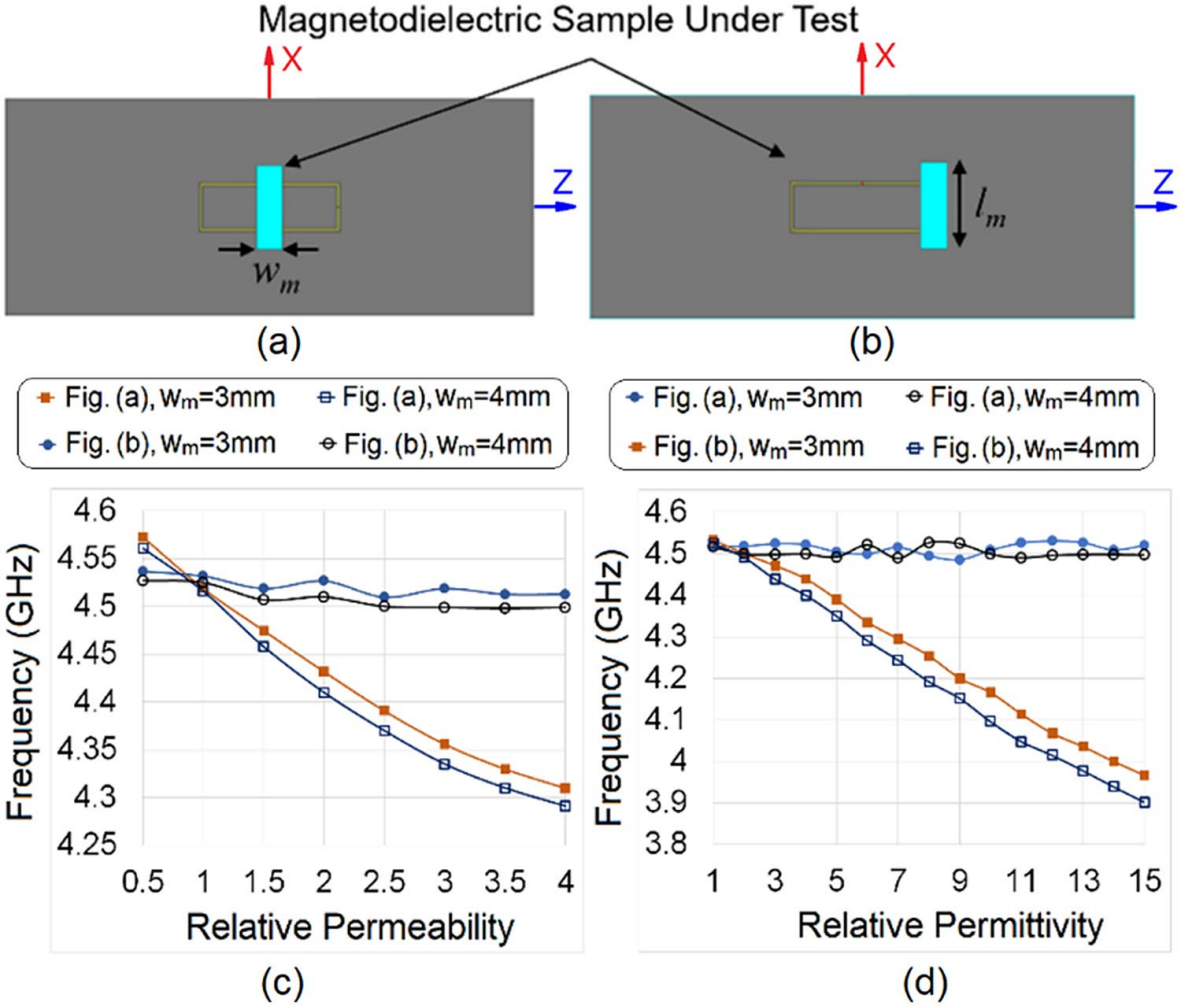
Sample placement for testing the (**a**) relative permeability and (**b**) relative permittivity of the magnetodielectric sample. Independent sensing profile of the proposed sensor under the loaded condition for various values of (**c**) relative permeability and (**d**) relative permittivity.[ANSYS Electromagnetics Suite, Release 2019 R2, https://www.ansys.com/products/electronics; and Microsoft Office Professional Plus 2019, https://www.office.com].

We found from Fig. 4 that the *y*-component of the magnetic field with uniform intensity and phase is available at the center of the rectangular patch at the ground plane, while the *x*-component of the electric field has high intensity at the side edges along the *z*-direction of the rectangular patch at z = (*a* ± *l*)/2. Therefore, the position z = *a*/2 can be used to perturb the magnetic field, while z = (*a* ± *l*)/2 can be used to perturb the electric field. The magnetodielectric sample under test (SUT), with a cross-section of *w*_*m*_ by *l*_*m*_, is shown in Fig. 5; the positions for magnetic testing and dielectric testing are depicted in Fig. 5a and b. For testing the order of control in the measurement of dielectric and magnetic properties, the SUT is systematically placed on the sensor following the procedure shown in Fig. 5a and b. The relative permittivity and relative permeability of the SUT vary over a wide range, from 1 to 15 and from 0.5 to 4, respectively. The length *l*_*m*_ and the height *w*_*h*_ of the SUT are kept fixed in this observation at 10 mm and 3 mm, while *w*_*m*_ is varied from 3 to 4 mm. The scattering parameters are recorded for each material loading. We observe that this structure exhibits a stopband response at the operating (resonant) frequency of the sensor (e.g., 4.5 GHz in unloaded condition), and the position of the resonance producing the stopband changes when the sensor is loaded. The output response is summarized and plotted in Fig. 5c and d. From Fig. 5c, the sensitivity to magnetic properties is found to be 75 MHz and 78 MHz per unit change of relative permeability for *w*_*m*_ = 3 mm and 4 mm, respectively, when placed in the configuration shown in Fig. 5a. From Fig. 5d, it can be observed that the sensor shows a sensitivity of 40 MHz and 45 MHz per unit change in relative permittivity for *w*_*m*_ = 3 mm and 4 mm, respectively, when placed in the configuration in Fig. 5b. It is interesting to note here that, for the same range of dielectric variation of SUT, when placed as in Fig. 5a, the maximum change in operating frequency is found to be less than the dielectric sensitivity of the sensor. A similar observation is seen in Fig. 5c: the fluctuation in operating frequency, when the SUT is placed as in Fig. 5b, is less than the sensitivity of the device for magnetic testing. This observation confirms that the sensor can independently test the dielectric and magnetic properties of the magnetodielectric SUT. It can also be seen that the device has better sensitivity in magnetic than in dielectric sensing. It is interesting to note that the proposed sensor can also detect the relative permeability of a SUT with either diamagnetic or paramagnetic characteristics (i.e., with relative permeability < 1 or ≥ 1). The MNZ sensor can thus be used to test a broad range of isotropic magnetodielectric materials at 4.5 GHz.

#### Volumetric analysis of dielectric and magnetic samples

**Figure 6:**
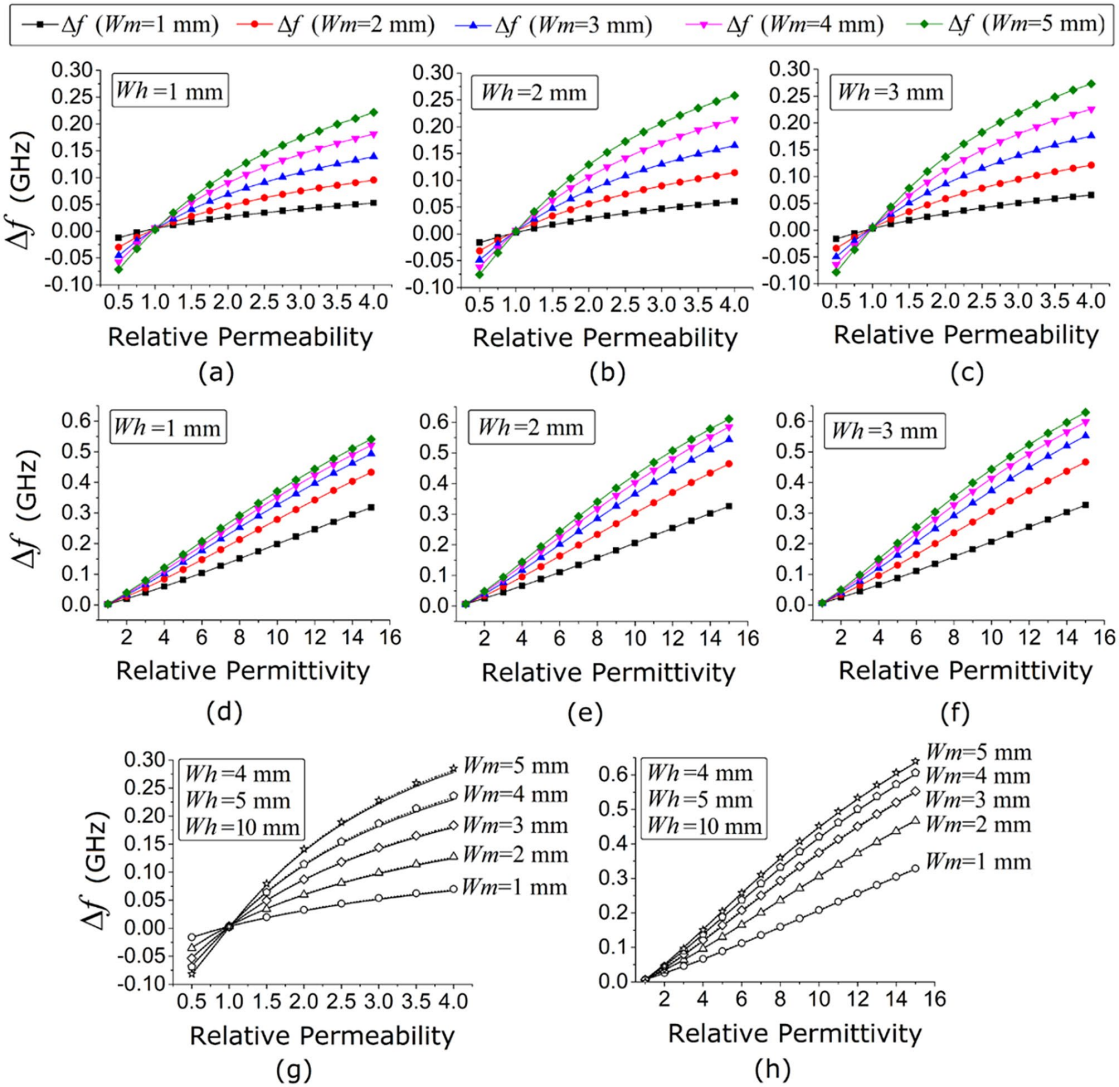
Change in relative frequency with respect to relative permeability of various SUTs with *w*_*m*_ = 1–5 mm, *l*_*m*_ = 10 mm and (**a**) *w*_*h*_ = 1 mm (**b**) *w*_*h*_ = 2 mm (**c**) *w*_*h*_ = 3 mm and (**g**) *w*_*h*_ = 4–10 mm; change in relative frequency with respect to relative permittivity of various SUTs with *w*_*m*_ = 1–5 mm, *l*_*m*_ = 10 mm and (**d**) *w*_*h*_ = 1 mm **e** *w*_*h*_ = 2 mm (**f**) *w*_*h*_ = 3 mm and (**h**) *w*_*h*_ = 4–10 mm.[OriginLab, 2020b, https://www.originlab.com].

Figure 5c and d demonstrate that the sensor has good control over independent measurements of the dielectric and magnetic properties of the magnetodielectric SUT. In the next step, we intend to examine the effect of the volumetric perturbation of the magnetodielectric sample. For this analysis, a variation of *w*_*m*_ from 1 to 5 mm and of *w*_*h*_from 1 to 10 mm are considered, while keeping *l*_*m*_ = 10 mm constant. For the magnetic and dielectric testing of SUT, the sample is loaded onto the sensor, as in Fig. 5a and b, and the relative permeability and relative permittivity are varied from 0.5 to 4 and from 1 to 15, respectively. We record the relative change in the resonant frequency (*Δf* = *f*_0_ − *f*_S_) for various magnetic and dielectric samples with a wide range of relative permeability, relative permittivity, and sample volume. The results are plotted in Fig. 6, where *f*_0_ and *f*_S_ are the unloaded and the loaded frequencies, respectively. Figure 6a–f present the profile of *Δf* with respect to the relative permeability and relative permittivity of a SUT with *w*_*m*_ ranging from 1 to 5 mm and *w*_*h*_ = 1 mm, 2 mm, and 3 mm, respectively. It can be observed from Fig. 6a–f that an increase in sample volume increases *Δf*, which is the result of an increase in the magnetic field and electric field perturbation, respectively. It is interesting to note from Fig. 6a–f that, irrespective of the values of relative permeability and relative permittivity, the increment in *w*_*h*_ increases the value of *Δf*; however, it can also be observed from Fig. 6g and h that increasing *w*_*h*_ by more than 4 mm does not have a significant effect on *Δf*. This is mostly due to the effect of the exponentially decaying near field in the vicinity of the rectangular patch on the ground. On the other hand, an increase in *w*_*m*_ from 1 to 5 mm always increases the value of *Δf*, where the maximum value of *w*_*m*_ is limited due to the fixed dimension of the rectangular patch.

## Sample characterization and sensor fabrication

**Figure 7:**
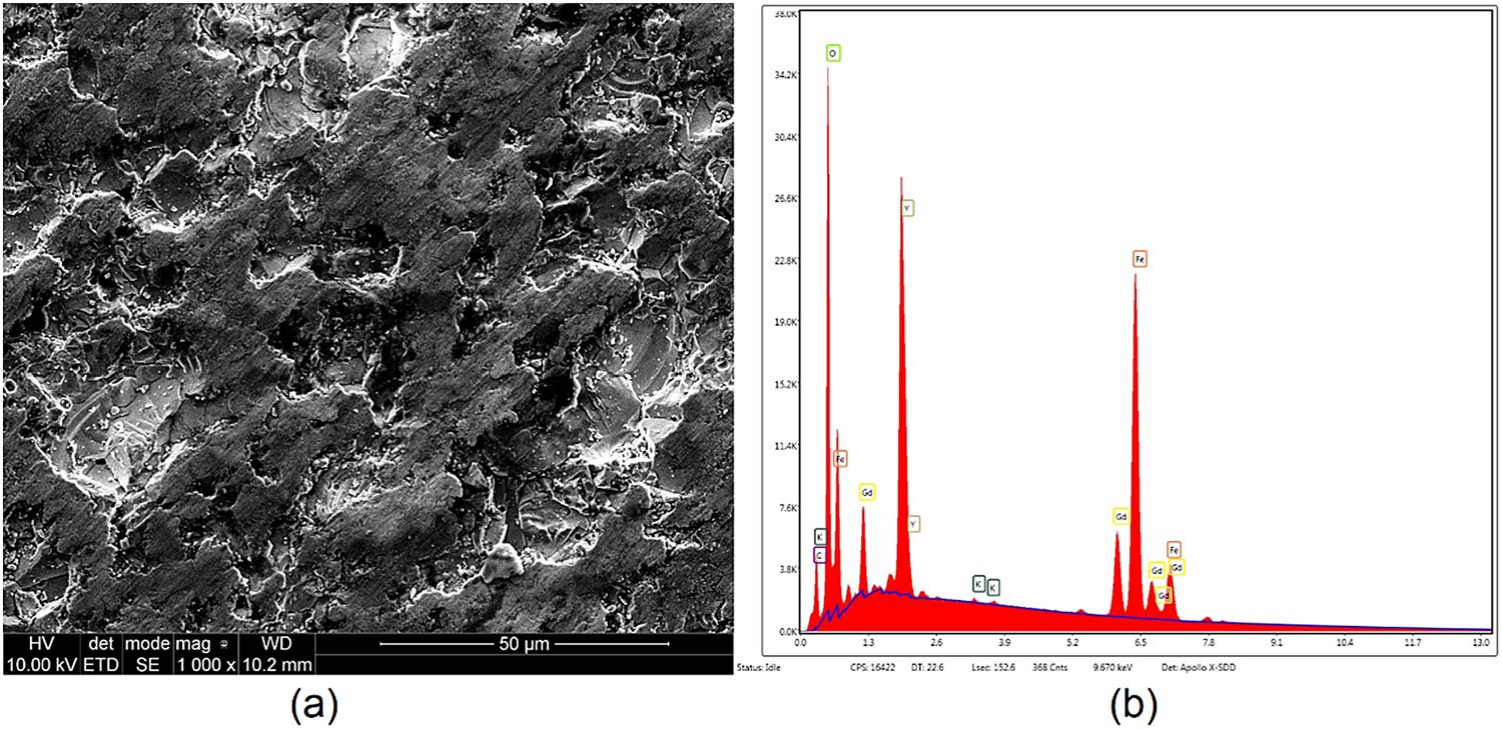
(**a**) scanning electron micrograph and (**b**) energy dispersive spectroscopy of the magnetodielectric sample [Quant 250 FEG (FEI), https://fei.com].

The dielectric and magnetodielectric samples were first fabricated. The dielectric samples were produced from standard microwave dielectric substrates: Taconic CER-10 (relative permittivity 10) and Rogers TMM13i (relative permittivity 12.2) by machine etching the metal from the top and bottom layers. The magnetodielectric sample was purchased from a local vendor and characterized by the scanning electron microscope Quanta 250 FEG (FEI) with an EDS analyzer (EDAX, Ametek). The surface morphology of the magnetodielectric sample is shown using micrographs in Fig. 7a, while the elementary energy dispersive spectroscopy (EDS) is shown in Fig. 7b. In Fig. 7a, a 1000× magnification at 50 μm confirms the minimum surface roughness for better contact with the sensing surface. From Fig. 7b, the EDS analysis confirms the empirical formulation to be GdY_2_Fe_5_O_12_, which suggests that the magnetic sample under test is a mixed yttrium gadolinium iron garnet^20–22^ Gd_x_Y_(3−x)_Fe_5_O_12_ with x = 1.

**Figure 8:**
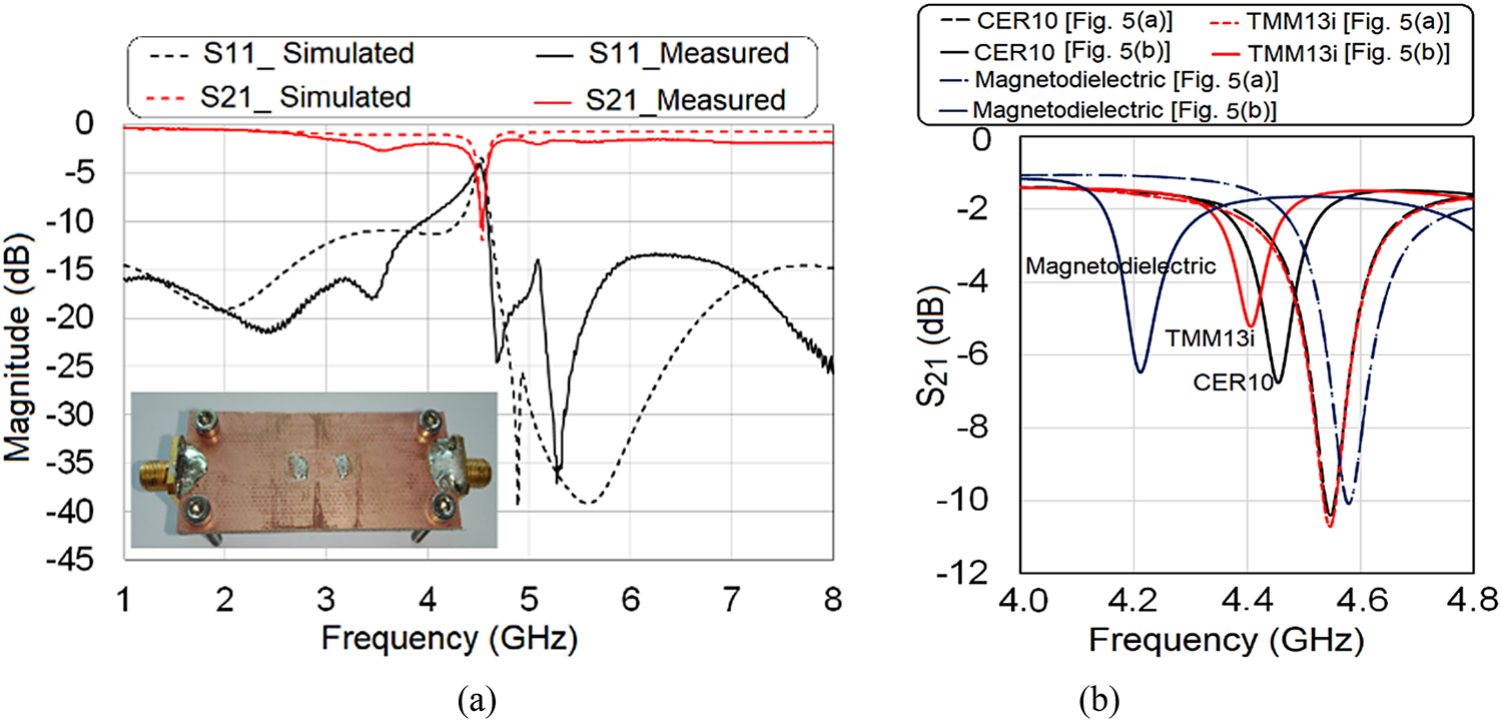
(**a**) Simulated and measured scattering parameters of the sensor and (**b**) measured response of dielectric and magnetodielectric SUTs.[OriginLab, 2020b, https://www.originlab.com].

The sensor was fabricated with the dimensions described in “Design of proposed double-layer sensor” section on a Taconic RF 35 substrate using an LPKF Protomat E33 milling machine. Two rows of three vias were drilled using the milling machine and metalized with LPKF ProConduct conductor polymer (conductivity ~ 1.5 × 10^5^ S/m). The two dielectric layers (layer1 and layer2) were combined using a thin layer of standard epoxy glue and were assembled using four 2.5 mm SEMS type round-head screws. Two female sub-miniature A (SMA) type connectors were mounted at the endpoints of the sensor to connect the scattering (S)-parameter measuring device. A photograph of the fabricated sensor is provided in the inset to Fig. 8a.

## Measurement and results

The vector network analyzer (an Agilent Technologies N5242A PNA-X network analyzer, 10 MHz–26.5 GHz) is calibrated using the electronic calibration kit. The fabricated sensor is connected to the vector network analyzer through a pair of coaxial cables, and the two-port S-parameters are measured in a wide band from 1 to 8 GHz. To determine the performance of the fabricated sensor, the measured S-parameters for an unloaded sensor are compared with the simulated S-parameters and shown in Fig. 8a, where the fabricated prototype is also shown in the inset. From Fig. 8a, it can be observed that the simulated and measured response have a good match, where the measured operating frequency (indicated by the notch in *S*_21_ due to resonance) is noted as 4.54 GHz. The dielectric samples, fabricated from the microwave substrates Taconic CER-10 (relative permittivity 10) and Rogers TMM13i (relative permittivity 12.2), have dimensions of 3 mm by 5 mm by 1.27 mm. The magnetodielectric sample GdY_2_Fe_5_O_12_ had dimensions of 3 mm by 10 mm by 5 mm. The details of the measurements around the resonant frequency are plotted in Fig. 8b. It is worth mentioning here that, when measuring the magnetic and dielectric properties, the dielectric substrates, and the magnetodielectric sample are loaded onto the sensor into the arrangement shown in Fig. 5a and b, respectively. It is interesting to observe from Fig. 8b that the dielectric substrates, when placed as in Fig. 5a, do not show significant shifts in resonant frequency, which confirms the non-magnetic response of the dielectric samples. Dielectric samples, when placed as in Fig. 5b, shows a different positive shift in resonant frequency. On the other hand, GdY_2_Fe_5_O_12_ shows a negative shift in resonant frequency, when loaded as in Fig. 5a, while the loading of GdY_2_Fe_5_O_12,_ shown in Fig. 5b provides a positive shift in the resonant frequency. Based on the volumetric analysis presented in “Volumetric analysis of dielectric and magnetic samples” section, quantitative analysis is performed to verify the accuracy of the proposed method; the results are given in Table 1. The measured values of relative permeability, and the relative permittivity of the SUT are compared with the reference values, and the error is computed and provided in Table 1.

**Table 1:** Measured Dielectric and Magnetic Properties of SUT.

Sample	Dimension	*Δf* when sample loaded as in Fig. 5a (MHz)	*Δf* when sample loaded as in Fig. 5b (MHz)	Relative permeability		Relative permittivity		Refs
				Measured value	Error (Δ)	Measured Value	Error (Δ)	
CER-10	3 mm × 5 mm × 1.27 mm	5.5	101	1.07	0.07	9.5	0.5	^23^
TMM13i	3 mm × 5 mm × 1.27 mm	6.1	140	1.09	0.09	11.6	0.6	^24^
GdY_2_Fe_5_O_12_	3 mm × 10 mm × 5 mm	− 30	330	0.67	0.17	9.2	–	^22^

## Measurement error analysis

**Figure 9:**
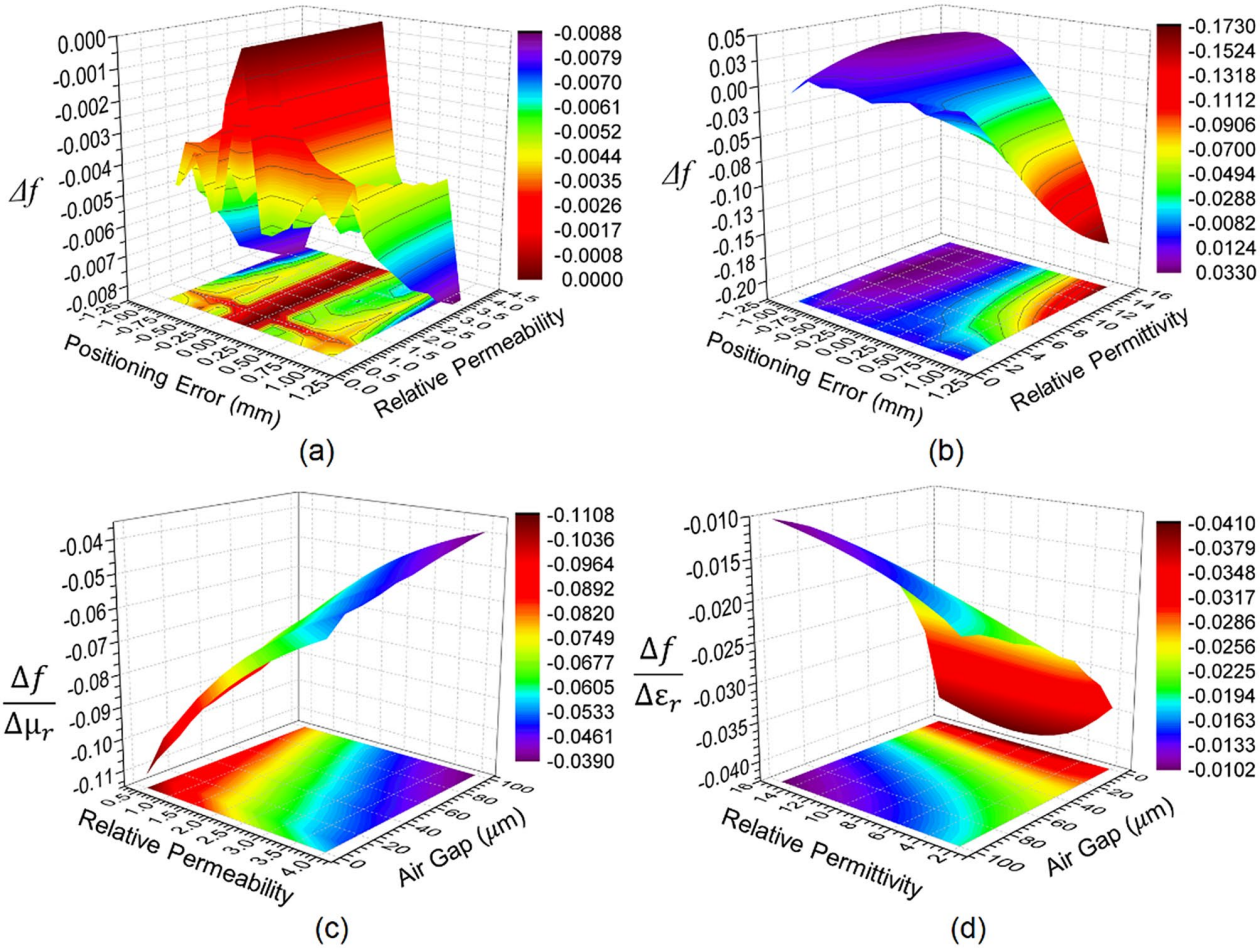
Relative changes in the resonant frequency due to positioning error for (**a**) magnetic SUTs and (**b**) dielectric SUTs; sensitivity profile due to air gap present between sensing layer and (**c**) magnetic SUT and (**d**) dielectric SUT.[OriginLab, 2020b, https://www.originlab.com].

Two types of errors are common while measuring SUTs in the microwave laboratory: positioning errors and air gap error. Due to the small dimensions of the sample, it is quite difficult to align the sample for each measurement following the arrangement shown in Fig. 5a and b. Error analysis is thus used to determine the variation in the practical measurement. When placing the solid sample on the ground plane, it can be difficult to eliminate the air gap between the SUT and sensor, leading to a need to carry out air gap error analysis also. The positioning error is estimated along the *z*-axis, taking z = *a*/2 as the reference line. The sample dimension is kept fixed for the two error analyses at 3 mm by 10 mm by 5 mm. A ± 1 mm error along the *z*-axis is assumed for the sample positioning error, while an air gap of up to 100 μm between the SUT and sensing area is assumed for the air gap error analysis. The two types of error are computed for a similar range of variation of permeability and permittivity and are provided in Fig. 9. Figure 9a and b represent the 3D mesh profile of the relative resonant frequency for the various magnetic and dielectric samples subjected to different positioning errors. It is interesting to note from Fig. 9a that a positioning fault of ± 0.25 mm leaves almost no trace on *Δf*, while increasing the positioning error by more than ± 0.25 mm rapidly decreases the value of *Δf.* However, the maximum value of *Δf* (8 MHz) for ± 1 mm positioning error introduces an error in the relative permeability equivalent to 9%. A similar observation on the basis of Fig. 9b suggests an entirely different response. While the positioning error of the magnetic sample is an even function, the dielectric sample is not. This difference in positioning error is attributed to the configuration of the magnetic field and the electric field of the sensor, as shown in Fig. 4. A negative positioning error in dielectric measurement produces positive *Δf,* while a positive positioning error gives a negative *Δf.*

From Fig. 9b, it can be observed that a ± 0.1 mm positioning error leaves almost no trace on *Δf*; however, 1 mm and − 1 mm positioning errors introduce shifts in the resonant frequency of 180 MHz and 33 MHz, respectively, producing a large error in the relative permittivity equivalent to 400% and 70%, respectively. It can be understood from the above analysis that the sensor is a good candidate for error-free measurement of the magnetic sample, thanks to the MNZ effect. Further, the air gap results are presented in terms of the sensitivity analysis for magnetic and dielectric samples in Fig. 9c and d. Sensitivity is calculated as the ratio of the change in relative permittivity to the change in relative permeability or relative permittivity. The general observation from these two figures suggests that increasing the air gap reduces the sensitivity of the measurement. However, a close examination of Fig. 9c indicates some unique behavior: relative permeabilities lower than one display some level of immunity to the sensitivity with respect to the air gap. Close inspection of Fig. 9d suggests another unique behavior: that a change in relative permittivity has almost no impact on the sensitivity when the air gap is less than 15 μm, which is absent in Fig. 9c. From Figs. 9c and d, we can also observe that the overall sensitivity in measuring the magnetic sample is higher than for the dielectric sample, thanks to the MNZ effect of the sensor.

## Conclusions

A novel MNZ sensor, the first of its kind, has been designed, analyzed, fabricated, and measured for the testing of dielectric and magnetodielectric materials. Detailed numerical calculations were performed to determine the electromagnetic field configuration, independent sensing control, performance analysis, positioning error, and air gap analysis. Two standard dielectric substrates and one yttrium gadolinium iron garnet were fabricated, characterized, and tested at 4.5 GHz. The results were found to be in good agreement with the literature. This sensor shows great potential in microwave device fabrication and in the microwave material industry.
